# Determination of Cadmium in Brown Rice Samples by Fluorescence Spectroscopy Using a Fluoroionophore after Purification of Cadmium by Anion Exchange Resin

**DOI:** 10.3390/s17102291

**Published:** 2017-10-09

**Authors:** Akira Hafuka, Akiyoshi Takitani, Hiroko Suzuki, Takuya Iwabuchi, Masahiro Takahashi, Satoshi Okabe, Hisashi Satoh

**Affiliations:** 1 Department of Integrated Science and Engineering for Sustainable Society, Faculty of Science and Engineering, Chuo University, Tokyo 112-8551, Japan; hafuka.14p@g.chuo-u.ac.jp; 2 Division of Environmental Engineering, Faculty of Engineering, Hokkaido University, Sapporo 060-8628, Japan; aaa.aki.zzz@gmail.com (A.T.); m-takaha@eng.hokudai.ac.jp (M.T.); sokabe@eng.hokudai.ac.jp (S.O.); 3 Department of Research and Development, Metallogenics Co., Ltd., Chiba 260-0856, Japan; hsuzuki@ak-j.com (H.S.); tiwabuchi@ak-j.com (T.I.)

**Keywords:** cadmium, rice, simple analytical method, fluorescence spectroscopy, pretreatment

## Abstract

Simple analytical methods are needed for determining the cadmium (Cd) content of brown rice samples. In the present study, we developed a new analytical procedure consisting of the digestion of rice using HCl, Cd purification using anion exchange resin, and then determining the Cd content using fluorescence spectroscopy. Digestion with 0.1 M HCl for 10 min at room temperature was sufficient to extract Cd from the ground rice samples. The Cd in the extract was successfully purified in preference to other metals using Dowex 1X8 chloride form resin. Low concentrations of Cd in the eluate could be determined using fluorescence spectroscopy with a fluoroionophore. Overall, the actual limit of quantification value for the Cd content in rice was about 0.1 mg-Cd/kg-rice, which was sufficiently low compared with the regulatory value (0.4 mg-Cd/kg-rice) given by the Codex Alimentarius Commission. We analyzed authentic brown rice samples using our new analytical procedure and the results agreed well with those determined using inductively coupled plasma optical emission spectrometry (ICP-OES). Since the fluoroionophore recognized Zn^2+^ and Hg^2+^ as well as Cd^2+^, a sample containing high concentration of Zn^2+^ or Hg^2+^ might cause a false positive result.

## 1. Introduction

In recent years, the contamination of agricultural land by heavy metals, such as cadmium (Cd) [1], mercury [2], chromium, copper (Cu), zinc (Zn) and lead [3] has become a major problem worldwide. This has increased the risks to food safety and hence human health because heavy metals can easily be absorbed from the soil by agricultural crops and then transferred into the human body through consumption. Among the heavy metals contaminating agricultural crops, Cd in rice is a great problem, especially in Asian countries [1,4,5]. Compared with other agricultural crops, rice tends to accumulate Cd readily [6] and thus can be a major source of dietary Cd intake for humans living in Asian countries where rice is a staple food [7]. Cd has toxic effects on humans leading to many serious diseases and some cancers [8]. In Japan, Itai-Itai disease occurred from the 1910s to the 1970s because rice, vegetables and drinking water had been contaminated with Cd [9]. Currently, China is also facing a similar situation [1]. In 2006, the Codex Alimentarius Commission set the international standard value of Cd contained in polished rice at 0.4 mg-Cd/kg-rice. Thus, a reliable method for determining the Cd content in rice samples has now become more important.

Currently, the most common analytical procedure for determining the Cd content of rice is a sample pretreatment followed by instrumental analysis using atomic absorption spectrometry (AAS), inductively coupled plasma optical emission spectrometry (ICP-OES), inductively coupled plasma mass spectroscopy (ICP-MS), atomic fluorescence spectrometry (AFS), or electrodes [10,11,12,13,14,15]. Although their sensitivity is high, these methods are time-consuming and require expensive instruments and complex operations. In contrast, simple analytical methods, based on colorimetry, ultraviolet–visible spectroscopy, and immunoassay have recently been developed to determine Cd in rice samples [16,17,18]. However, these methods lack a simple pretreatment, have low sensitivity, and are not yet adequate for application to real samples. Fluorescence spectroscopy is an alternative method which has attracted a great deal of attention because of its high sensitivity, simplicity, and versatile instrumentation [19]. Like the other simple methods mentioned above, determining Cd using fluorescence spectroscopy depends largely on the characteristics of the indicator used (e.g., a fluoroionophore)—its sensitivity, selectivity, photo-physical properties, and water solubility. Because no indicator has perfect selectivity towards the target analyte, samples must be purified before determining the Cd content. Zhang et al. have determined the Cd^2+^ content in rice samples using a solid phase extraction (SPE)-assisted fluorometric paper sensor [20]. They purified and preconcentrated the Cd^2+^ using SPE then determined its content using a fluoroionophore immobilized on the test paper. For this method, the limit of detection (LOD) was poor (56 μg-Cd/L-solution) and the digestion method used for pretreating the rice, consisting of microwave irradiation with mixed acid solution, was complicated. Therefore, we have proposed a new analytical procedure consisting of rice digestion, Cd purification, followed by Cd determination using fluorescence spectroscopy as a simple method of analysis for Cd in rice. In the present study, we aim to develop a simple digestion method using 0.1 M HCl, a Cd purification method using anion-exchange resin, then the ratiometric determination of Cd using fluorescence spectroscopy.

## 2. Materials and Methods 

### 2.1. Standard Methods of Rice Digestion and Metal Determination

Brown rice samples were obtained from rice farmers. The brown rice standard sample (NMIJ CRM 7531-a) was purchased from the National Metrology Institute of Japan (Tsukuba, Japan). Milli-Q water (18.2 MΩ·cm) was used in all experiments described below. Rice digestion and metal determination were conducted based on Japanese standard method (Japanese Ministry of Health). Briefly, a sample of ground rice (10 g) was added to 10.4 M of HNO_3_ (50 mL) then the suspension was gently heated. The suspension was cooled to room temperature then concentrated H_2_SO_4_ (2 mL) was added. The suspension was heated again until its color changed to light yellow or colorless. After cooling to room temperature, the solution was transferred to a volumetric flask (100 mL) and 0.1 M HNO_3_ was made up to the mark. The concentrations of Cd, Zn, Cu and Iron (Fe) were determined using an ICP-OES instrument (ICPE-9000, Shimadzu Corporation, Kyoto, Japan).

### 2.2. Hydrochloric Acid Digestion of Rice Samples

Rice samples (20 g) were ground for 10 s using a laboratory-scale mill (Labo Milser LM-PLUS, Osaka Chemical Co., Ltd., Osaka, Japan). The sample powder was then added to 0.1 M HCl (80 mL) and the mixture stirred at room temperature for 10 min. Following centrifugation at 8000 g for 10 min, the supernatant was filtered through a 1.0-μm-pore-size cellulose ester membrane (A100A047A, Advantec Toyo Kaisha Ltd., Tokyo, Japan) to obtain the extract. The extract solution (9 mL) and concentrated HNO_3_ (1 mL) were added in 10 mL-test tubes then ICP-OES measurements were conducted. The Cd concentration of the extract determined using ICP-OES was compared with that determined by the standard method to evaluate the extraction efficiency.

### 2.3. Purification of Cd by Anion-Exchange Resin

We adopted the method by Kallmann et al. [21] to purify Cd from other metals. It uses the difference in stability between the negatively-charged chloro-complex of Cd and other metals in a HCl solution (Cd chloro-complex is more stable than the chloro-complexes of other metals) and separates Cd from other metals using an anion-exchange resin.

Dowex 1X8 chloride form (44340, Sigma-Aldrich Japan K.K., Tokyo, Japan) was used as the anion-exchange resin. The resin was washed three times with Milli-Q water (10 times the volume of the resin) then soaked in Milli-Q water overnight. A disposable polypropylene column (5 mL; 29922, Thermo Fisher Scientific K.K., Yokohama, Japan) held the resin. The column was washed with Milli-Q water then filled with 10% ethanol to obtain the surface hydrophilicity of the column. A frit was introduced at the bottom of the column then 0.5 mL of the prepared resin was added to give a column volume (CV) of 0.5 mL. Finally, the resin was capped with another frit then washed with Milli-Q water (20 mL) to remove the ethanol completely. The prepared columns were preserved at 4 °C in a refrigerator before use. 

For the Cd purification process, water (2 mL) and 0.1 M HCl (5 mL) were passed through the column sequentially before adding the sample. The rice extract solution (40 mL = 80 CV) was passed through the column and the flow-through fraction was obtained. Then, 0.1 M HCl (40 mL = 80 CV) was passed through the column to remove organic compounds and metals (except Cd) to obtain the washout fraction. Finally, water (30 mL = 60 CV) was passed through the column and the Cd was extracted in the elution fraction. The Cd, Zn, Cu, and Fe concentrations of the flow-through, washout and elution fractions were determined using ICP-OES to estimate the elution efficiency.

### 2.4. Determination of Cd by Fluorescence Spectroscopy

**Figure 1:**
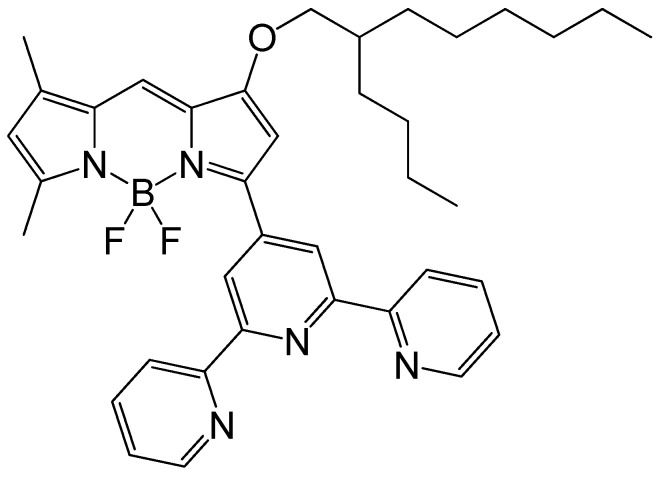
Chemical structure of 2,2′:6′,2″-terpyridine-substituted BODIPY (BDP-TPY).

The fluoroionophore, 2,2′:6′,2′′-terpyridine-substituted BODIPY (BDP-TPY), designed in our laboratory, was synthesized according to a previously reported method [22]. The chemical structure of BDP-TPY is shown in Figure 1. Since 2,2′:6′,2′′-terpyridine (TPY) acts as a heavy metal ion receptor, the fluorescence color of BDP-TPY changes upon binding of heavy metals to TPY [22]. A stock solution of BDP-TPY (2 μM) was prepared in acetonitrile. The test solutions were prepared by adding BDP-TPY stock solution (5 mL) to a volumetric flask (10 mL), followed by the elution fraction (4.5 mL) and 80 mM Tris buffer (0.5 mL, pH 10.2). The solutions were then transferred to quartz cells and the fluorescence spectra recorded by a spectrofluorometer (FP-6600, JASCO Corporation, Tokyo, Japan). The Cd concentrations of the elution fraction obtained were compared with those determined using ICP-OES. 

## 3. Results and Discussion

### 3.1. Acid Digestion of Rice Samples

**Figure 2:**
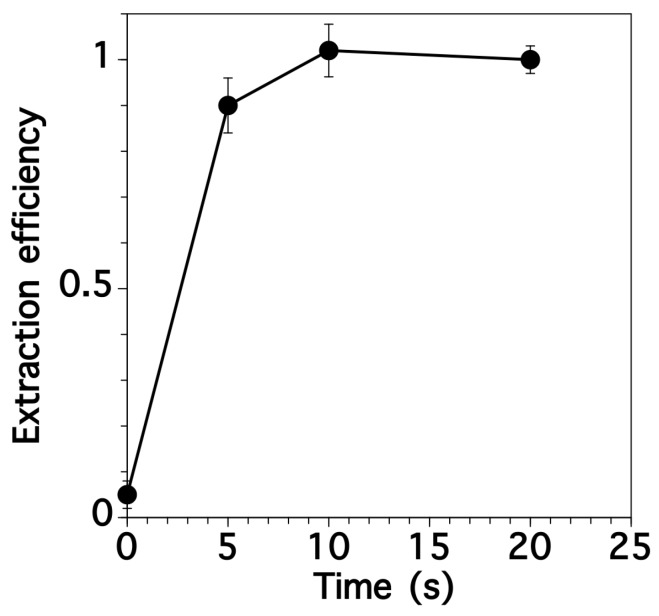
Extraction efficiency of Cd from brown rice samples as a function of grinding time. Error bars indicate the standard deviation, which was determined using three replicates.

Rice samples require acid digestion before the metal contents can be determined. In the first step, we optimized the time for grinding the samples. Figure 2 shows the efficiency of extracting Cd from a brown rice sample as a function of grinding time. Standard methods (Section 2.1) were used to digest the rice and determine the metal content. Around 90% of the Cd content was extracted after only 5 s grinding. After 10 s, the extraction efficiencies for Cd reached 100% so this was selected. Therefore, we set grinding time as 10 s for the subsequent experiments. The samples could be ground into a fine powder after 10 s of grinding using a laboratory-scale mill.

**Figure 3:**
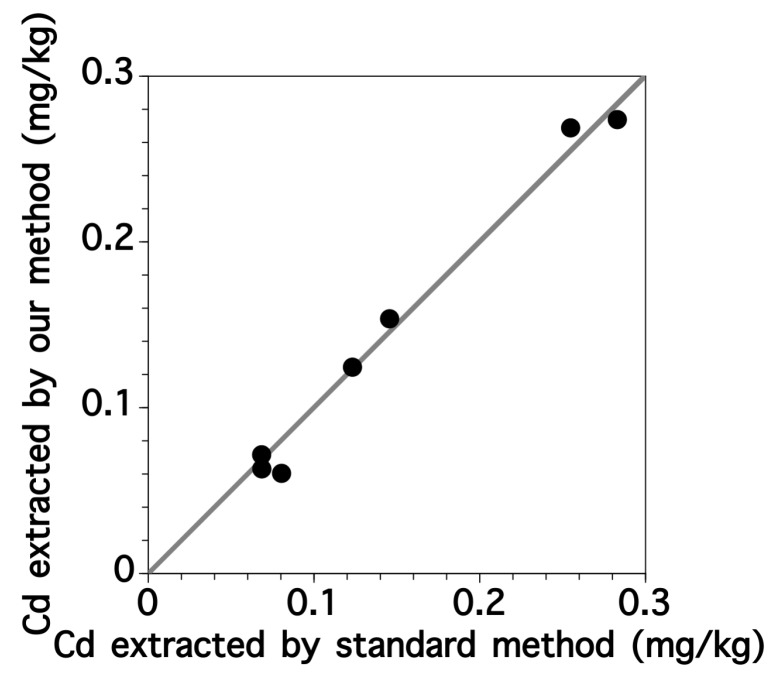
Relationship between Cd concentrations extracted by the standard (ICP-OES) and our developed methods.

The standard method for rice digestion is complicated and time-consuming. Therefore, we developed a simpler method with fluorescence spectroscopy using 0.1 M HCl solution (Section 2.2) which was appropriate for the following purification and fluorescence determination steps [22]. The Cd concentrations in the rice extracts were determined by the standard and our methods (Figure 3). There was an excellent correlation with a determination coefficient of 0.996 between the concentrations determined by the two different methods. This indicated that our simple digestion method was adequate for extracting Cd from the ground rice samples. After 10 min of HCl treatment, the fine rice powder became sticky.

### 3.2. Metals in Column Fractions

**Figure 4:**
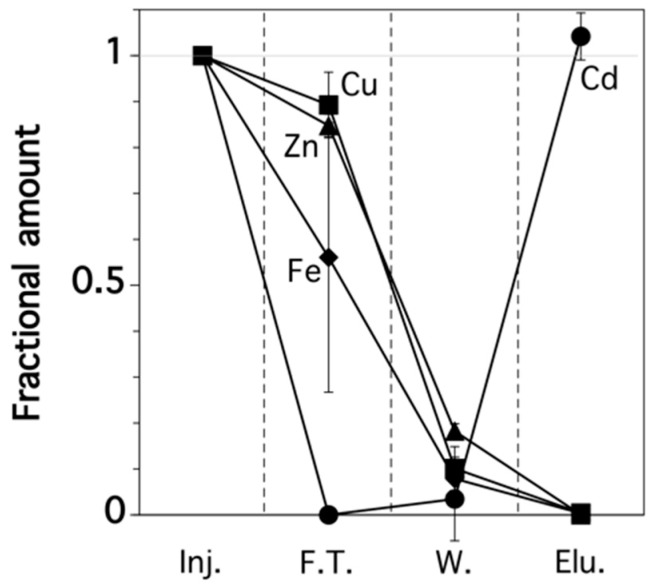
Relative amounts of Cd, Zn, Cu, and Fe from a real rice sample in each column fraction. Inj.: injection; F.T.: flow-through fraction; W.: washout fraction; Elu.: elution fraction. Error bars indicate the standard deviation, which was determined using three replicates.

The rice extract solutions were run through a polypropylene column packed with anion-exchange resin to purify the Cd. As well as the Cd concentration, we determined the Zn, Cu, and Fe concentrations in each column fraction because these other metals may inhibit the determination of Cd when using fluorescence spectroscopy [23]. Figure 4 shows the fractional amount of these four metals. In the first step, the negatively-charged chloro-complex of Cd formed during the extraction process was ion exchanged with Cl^−^ so that the complex was retained on the resin. In contrast, most of the other metals, which were in the free ionic form, passed through the column and collected in the flow-through fraction. During the washout step, 0.1 M HCl solution was passed through the column to remove any residual Zn, Cu, Fe, and organic compounds, especially soluble starch, into the washout fraction. During the elution step, Cd^2+^ was eluted from the column by passing through Milli-Q water. H_2_O might exchange chloride anion in the chloro-complex of Cd and convert the Cd complex to free Cd ion, which was then washed out by leaving chloride anion on the column. This was collected in the elution fraction because of the decomposition of the chloro-complex form, which was then analyzed during the following fluorescence determination step. It should be noted that the Cd was purified and its concentration was concentrated 1.33 times during these processes.

**Figure 5:**
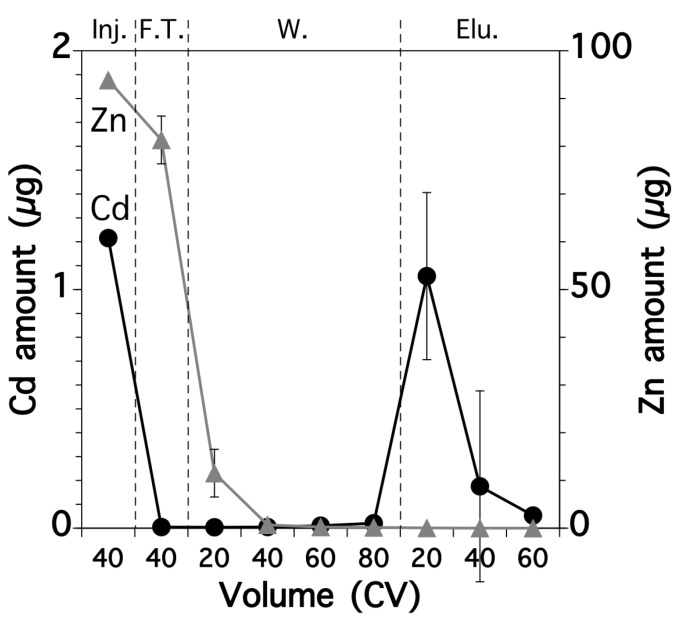
Amounts of Cd and Zn from a real rice sample in each CV of each column fraction. Inj.: injection; F.T.: flow-through fraction; W.: washout fraction; Elu.: elution fraction. Error bars indicate the standard deviation, which was determined using three replicates.

We determined the Cd and Zn contents in each fraction because Zn can strongly inhibit the determination of Cd using BDP-TPY [23]. The flow-through fraction contained most of the Zn (87%) and the first 20 CV of the washout fraction the remaining Zn content (Figure 5). Cd was rarely detected in the flow-through and washout fractions. Most of the Cd was found in the first 20 CV of the elution fraction and the residual Cd was recovered in the later elution fractions.

### 3.3. Determination of Cd by Fluorescence Spectroscopy

**Figure 6:**
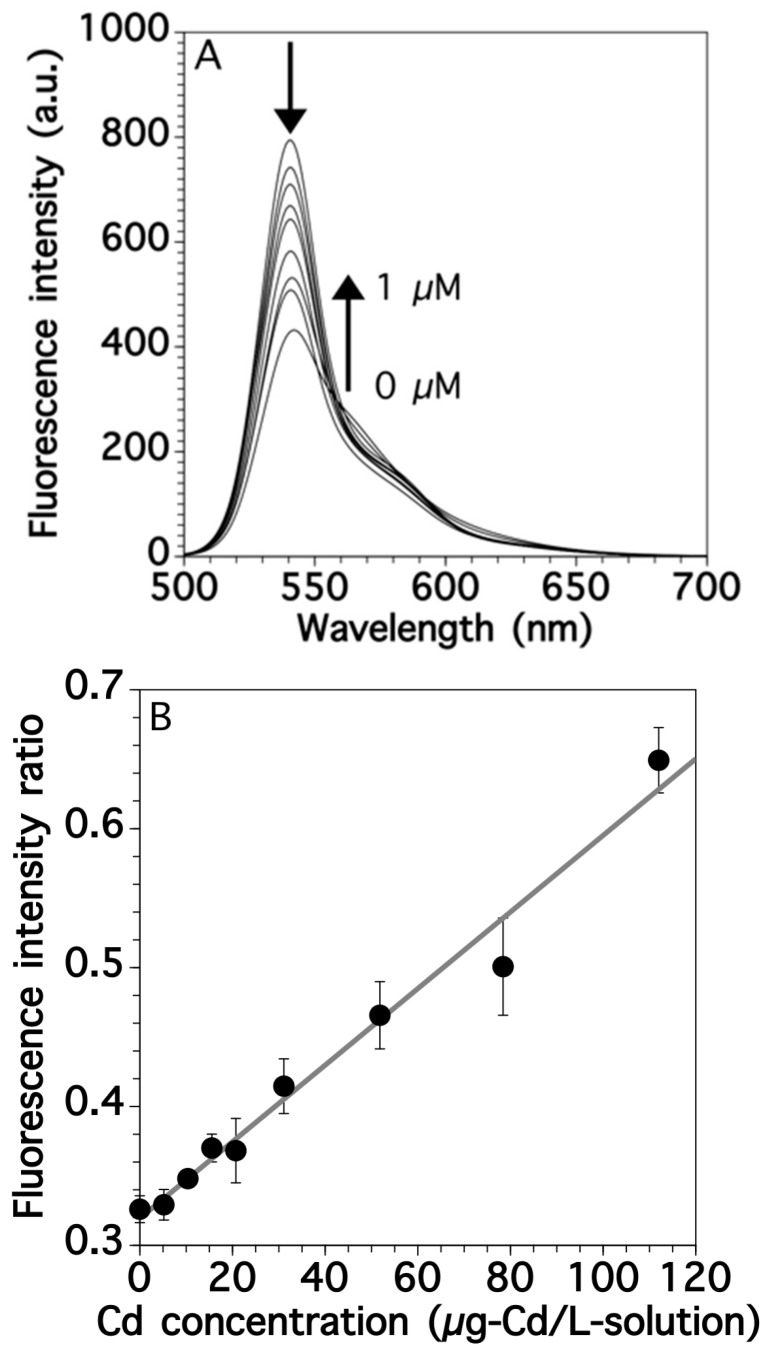
(**A**) Spectra and (**B**) calibration curve for Cd by fluorescence spectroscopy. Error bars indicate the standard deviation, which was determined using three replicates.

We established a calibration curve for Cd by fluorescence spectroscopy with BDP-TPY. Since the fluorescence spectra of BDP-TPY can be affected by pH value due to the protonation reaction [23], fluorescence titration experiments of BDP-TPY with Cd to generate a calibration curve were carried out in Tris buffer. BDP-TPY had a sharp fluorescence peak at 539 nm and its spectra changed as the Cd concentrations of the standard solutions increased. The height of the original fluorescence peak at 539 nm (F539) gradually decreased then a new fluorescence peak at 562 nm (F562) appeared and increased because of the transfer of intramolecular charges (Figure 6A) [22]. This shift in the spectra allowed the ratiometric measurement of Cd by calculating the fluorescence intensity ratio (F539/F562) [24]. The ratiometric measurement has advantages, such as, independency of results on probe concentration, bleaching, optical path length, illumination intensity, etc. F539/F562 increased linearly with Cd concentrations up to 112 μg-Cd/L-solution (1 μM) (Figure 6B), with the regression equation of y = 0.0027x + 0.32 (R^2^ = 0.978). The LOD (3s/slope) and limit of quantification (LOQ, 10 s/slope) values for Cd were determined based on the standard deviations of 11 blank solutions. The LOD and LOQ values were estimated to be 3.8 μg-Cd/L-solution (0.03 μM) and 12.7 μg-Cd/L-solution (0.11 μM), respectively. These values were relatively low compared with those of simple analyses previously reported [18,20]. Furthermore, a dynamic range of BDP-TPY was investigated. The linear range of Cd was up to 224 μg-Cd/L-solution (2 μM) (Figure S1 in the Supplementary Material of this paper).

Since the elution fraction of the rice extract solutions was acidic, we had to optimize the composition of the buffer solution for the fluorescence determination. Figure S2 in the Supplementary Material of this paper shows the effects of Tris concentrations on the pH of the analytical samples. The pH values of the elution fraction were acidic after the addition of 20 and 60 mM Tris buffer. Above 70 mM, the pH was approximately neutral so we decided to use 80 mM Tris buffer for the fluorescence determination.

**Figure 7:**
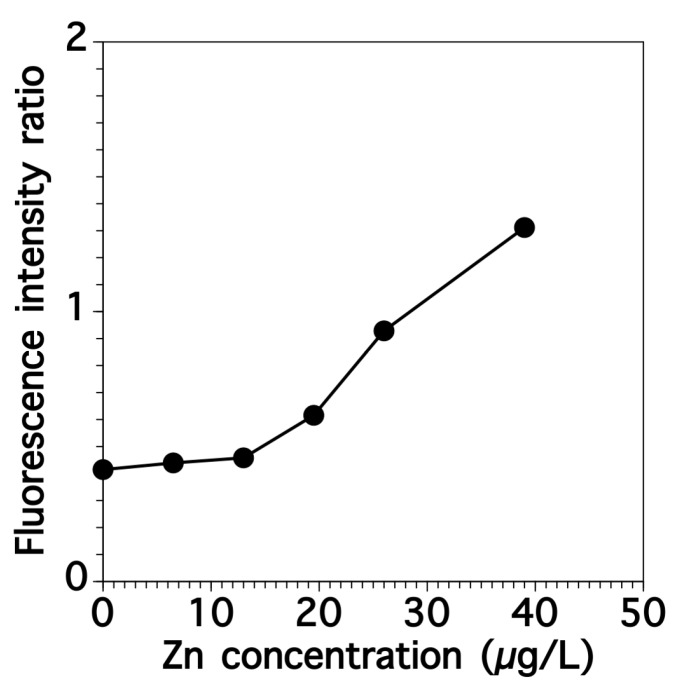
Effect of Zn concentrations on fluorescence intensity ratio (F539/F562) in the sample containing Cd (0.3 μM).

We investigated the interfering effect of Zn on Cd determination using fluorescence spectroscopy. Figure 7 shows the relationship between the fluorescence intensity ratio and the Zn concentration while the Cd concentration was kept constant at 33.6 μg-Cd/L-solution (0.3 μM). The ratios gradually increased and an interfering effect was observed at Zn concentrations above 19.5 μg-Zn/L-solution (0.3 μM). Although no Zn was detected in the elution fraction, the Zn concentration should be kept below 19.5 μg-Zn/L-solution when using this method.

**Figure 8:**
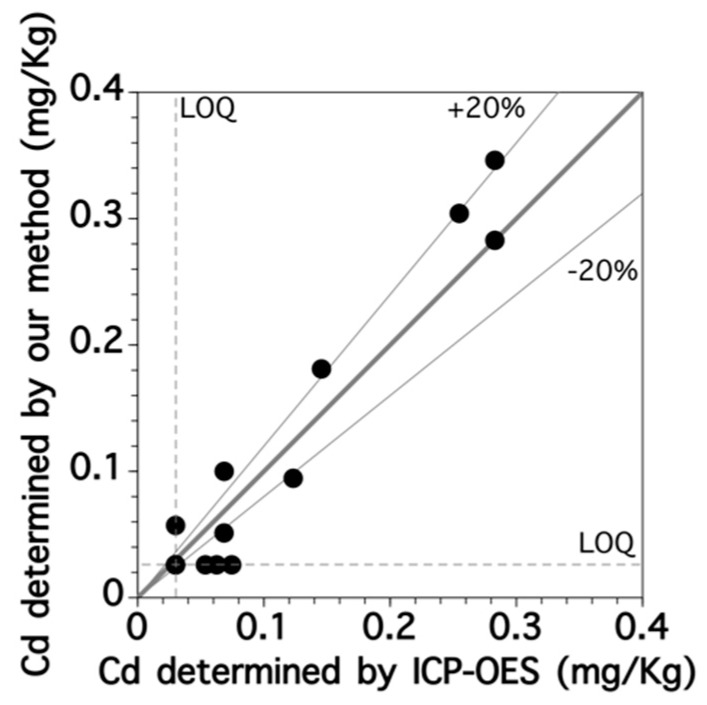
Relationship between concentrations of Cd in brown rice samples determined by fluorescence spectroscopy with the fluoroionophore and by ICP-OES.

We then investigated the applicability of fluorescence spectroscopy for measuring the Cd content of rice samples both by the standard method using ICP-OES and by our procedure consisting of HCl digestion, column purification and fluorescence spectroscopy. The Cd contents of fifteen brown rice samples were determined using the standard and our methods (Figure 8), revealing an almost linear relationship with a slope of 1.09 and a determination coefficient of 0.964. For most samples, the relative errors in the Cd contents determined by these two methods were within 20%. We thus concluded that Cd contents could be successfully determined in rice samples with complex matrices using our developed procedure. Overall, the actual LOQ value of the Cd content in brown rice was about 0.1 mg-Cd/kg-rice based on the results shown in Figure 8 Since the calibration error exceeded 20% of the calibration span value below 0.1 mg-Cd/kg-rice of Cd contents in brown rice. These values were sufficiently low compared with the regulatory value (0.4 mg-Cd/kg-rice) given by the Codex Alimentarius Commission. Therefore, our method would be suitable for the simple screening of the Cd content in rice samples.

## 4. Conclusions

In the present study, we have developed a simple analytical procedure for determining the Cd content of brown rice samples. We have revealed that digesting a rice sample with 0.1 M HCl for 10 min at room temperature effectively extracted Cd from a ground rice sample. The Cd in the extract was successfully purified in the presence of Zn, Cu, and Fe using a Dowex 1X8 chloride form resin. The Cd content in the elution fraction could be determined using fluorescence spectroscopy with a fluoroionophore with LOD and LOQ values of 3.8 μg-Cd/L-solution and 12.7 μg-Cd/L-solution, respectively. We analyzed authentic brown rice samples using our developed method and the results agreed well with those measured using ICP-OES. Overall, the actual LOQ value for Cd content in brown rice was about 0.1 mg-Cd/kg-rice. Based on these results, we concluded that our developed method would be suitable for the simple screening of the Cd content in rice samples. Nevertheless, as we reported previously, BDP-TPY is a fluoroionophore with high selectivity for Zn, Cd and Hg ions [22]. We also revealed that a chemical structure of an ion receptor of the BODIPY derivative determined the selectivity of it [22] and substitution at the 5-position of an asymmetric BODIPY cation sensor affected the selectivity of it [25]. Based on these results, at present we are trying to develop a novel BODIPY derivative with high selectivity for Cd.

## Supplementary Materials

The following are available online at http://www.mdpi.com/1424-8220/17/10/2291/s1. Figure S1: Calibration curve for Cd by fluorescence spectroscopy, Figure S2: Effect of Tris concentration of the buffer solution on the pH of the solution under analysis.
