# Supplementary material for: Determination of Cadmium in Brown Rice Samples by Fluorescence Spectroscopy Using a Fluoroionophore after Purification of Cadmium by Anion Exchange Resin
Source: Sensors (Basel). 2017 Oct 9;17(10):2291. doi: 10.3390/s17102291 (PMC5677403; doi:10.3390/s17102291)
Supplement: Supplementary file 1 [file sensors-17-02291-s001.pdf]

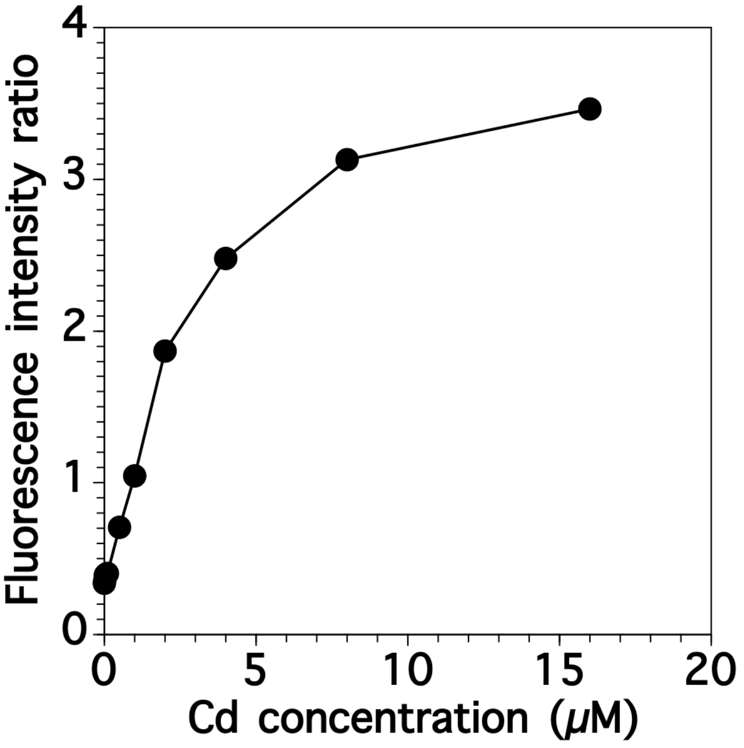

Figure S1. Calibration curve for Cd by fluorescence spectroscopy.

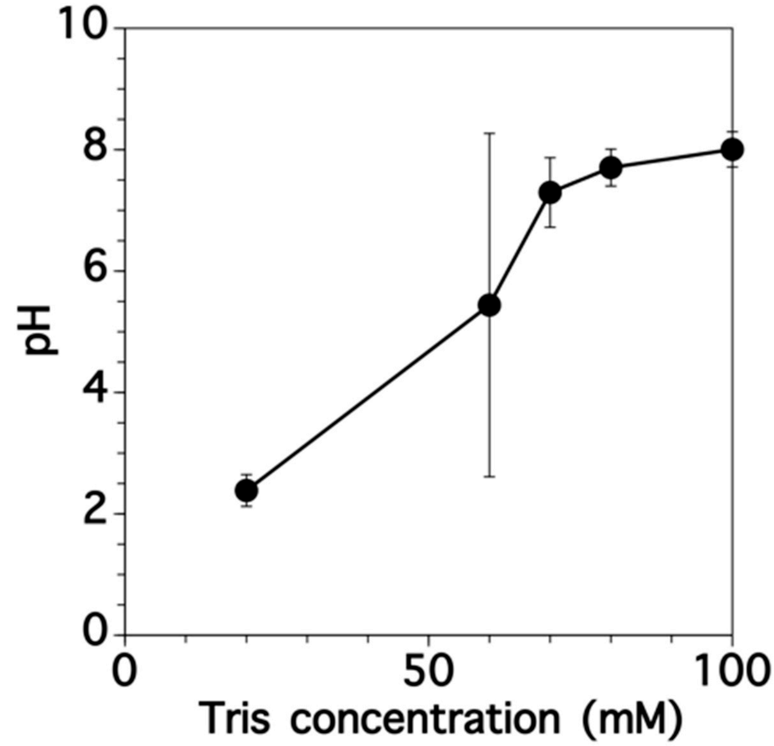

Figure S2. Effect of Tris concentration of the buffer solution on the pH of the solution under analysis.
